# Development of a NIR Method for the In-Line Quantification of the Total Polyphenolic Content: A Study Applied on *Ajuga genevensis* L. Dry Extract Obtained in a Fluid Bed Process

**DOI:** 10.3390/molecules23092152

**Published:** 2018-08-27

**Authors:** Alexandru Gavan, Liora Colobatiu, Andrei Mocan, Anca Toiu, Ioan Tomuta

**Affiliations:** 1 Department of Medical Devices, Iuliu Hatieganu University of Medicine and Pharmacy, 4 Louis Pasteur Street, Cluj-Napoca 400439, Romania; gavan.alexandru@umfcluj.ro; 2 Department of Pharmaceutical Botany, Iuliu Hatieganu University of Medicine and Pharmacy, 23 Gheorghe Marinescu Street, Cluj-Napoca 400337, Romania; mocan.andrei@umfcluj.ro; 3 Department of Pharmacognosy, Iuliu Hatieganu University of Medicine and Pharmacy, 12 Ion Creanga Street, Cluj-Napoca 400010, Romania; atoiu@umfcluj.ro; 4 Department of Pharmaceutical Technology and Biopharmacy, Iuliu Hatieganu University of Medicine and Pharmacy, 41 Victor Babes Street, Cluj-Napoca 400012, Romania; tomutaioan@umfcluj.ro

**Keywords:** *Ajuga genevensis*, near-infrared spectroscopy, dry extract, fluid bed process, microNIR, in-line monitoring, total polyphenolic content

## Abstract

This study describes an innovative *in-line* near-infrared (NIR) process monitoring method for the quantification of the total polyphenolic content (TPC) of *Ajuga genevensis* dry extracts. The dry extract was obtained in a fluidized bed processor, by spraying and adsorbing a liquid extract onto an inert powder support. NIR spectra were recorded continuously during the extract’s spraying process. For the calibration of the *in-line* TPC quantification method, samples were collected during the entire process. The TPC of each sample was assessed spectroscopically, by applying a UV-Vis reference method. The obtained values were further used in order to develop a quality OPLS prediction model by correlating them with the corresponding NIR spectra. The final dry extract registered good flowability and compressibility properties, a concentration in active principles three times higher than the one of the liquid extract and an overall process yield of 85%. The average TPC’s recovery of the NIR in-line prediction method, compared with the reference UV-Vis one, was 98.7%, indicating a reliable monitoring method which provided accurate predictions of the TPC during the process, permitting a good process overview and enabling us to establish the process’s end point at the exact moment when the product reaches the desired TPC concentration.

## 1. Introduction

The genus *Ajuga* L., from the family Lamiaceae, includes over 300 species of annual and perennial herbs in global distribution [1,2]. A number of six *Ajuga* species are mentioned in the spontaneous Romanian flora, these being mainly used in our traditional medicine due to their anti-inflammatory, wound healing, hepatoprotective, and anti-diarrheal pharmacological properties [3,4].

Phytochemical studies revealed the presence of multiple bioactive compounds in *Ajuga* species, including: phytoecdysteroids, *neo*-clerodane-diterpenes and diterpenoids, triterpenes, sterols, anthocyanidin-glucosides and iridoid glycosides, flavonoides, triglycerides, tocopherol and essential oils [3,5,6,7]. However, the active compounds of the same plant species may be different from one region to another, in terms of chemistry, pharmacology and toxicology. There is little information on the chemical composition of Romanian *Ajuga* species, only *A. reptans*, *A. genevensis* and *A. laxmannii* being previously analyzed [3,5,7,8].

In general, phytomedicines require exceptional chemical and physical stability, as well as low microbial growth in order to be used in therapy. Therefore, dry powder extracts are among the most suitable pharmaceutical forms of phytomedicines, due to their characteristics in the solid state, in addition to the possibility of easy preparation of tablets and capsules [9,10,11].

The fluid bed process is mostly used for drying and granulation purposes, being frequently an indispensable step in solid oral dosage form manufacturing. It usually consists in obtaining granules by spraying a binder solution over a fluidized powder bed [12]. However, the fluidized bed process can be also used as a fast method in order to obtain dry extracts by spraying and adsorbing liquid herbal hidroalcoholic extracts over a solid support [13].

The increasing demand for quality in herbal medicines has prompted a dire need for the quantitative analysis of bioactive compounds (including polyphenols) in herbs or medicinal plant extracts or herbal products by the use of efficient analytical methods [14].

Polyphenolic compounds are widely known today as antioxidant, antimicrobial, antiviral and anti-inflammatory agents, becoming desirable plant metabolites and being frequently used in the treatment of specific pathological conditions [15,16,17,18].

Different analytical methods such as high performance liquid chromatography (HPLC), gas chromatography (GC) or combinations of these analyses with mass spectrometry (MS) are currently employed in the determination of phenolic compounds [3,19,20,21,22]. Even though efficient, for they provide rapid separation and quantification of phenolic compounds, these techniques also require a lot of pre-processing steps before and during the analysis [17,22].

In recent years, the near-infrared (NIR) spectroscopy, especially combined with chemometric algorithm, has become an extensively used tool due to its fast, non-destructive, and low-cost characteristics, being widely applied in many unit operations of pharmaceutical manufacturing, including blending, granulation, fluidized bed drying, tablet-compression and coating, etc., [23]. NIR can be used *at-line* (measurement where the sample is removed, isolated from, and analyzed in close proximity to the process stream), *on-line* (measurement where the sample is diverted from the manufacturing process and may be returned to the process stream), as well as *in-line* (measurement where the sample is not removed from the process stream) [24]. Techniques based on NIR spectrometry have been used in fluidized bed granulation processes in order to determine the end-point of the process, as well as to monitor particle size and more frequently, moisture content (as water molecules strongly absorb NIR emissions on the 1400 and 1900 nm region) [23,25,26,27]. It has also been applied for the *in-line* quantification of film thickness of pharmaceutical pellets during fluid bed coating processes [28].

Recently, NIR spectroscopy (coupled with multivariate analyses) has also found excellent potential for quick and inexpensive quantification of total polyphenolic content (TPC) [29]. The technique has been recently applied (either *at-line* or *on-line*) to determine the content of phenolic compounds in yerba mate [22], the total content of emodin, chrysophanol, rhein, aloeemodin, and physcion in *Rhei Radix et Rhizoma* [30], for the simultaneous testing of total polyphenols, caffeine and free amino acids in Chinese tea from different categories [31], for measuring the phenolic composition of propolis [32], as well as for the total polyphenols quantification in *Acridocarpus orientalis* and *Moringa peregrina* [17].

Although, until now, *in-line* NIR spectroscopy technology has been mainly applied in the fluidized bed processes in order to monitor parameters such as particle size or moisture content, to the best of authors’ knowledge, no such method has been yet developed in order to assess the TPC from dry extracts [23,33,34].

The aim of this study was to develop a manufacturing method for obtaining dry *A. genevensis* extracts by implementing a fluidized bed process, as well as to develop an innovative *in-line* NIR method for the quantification of their TPC.

## 2. Results and Discussion

The concentration of total polyphenols in the initially obtained liquid *A. genevensis* herbal extract was 1.88%. The liquid extract was further used in the fluidized bed preparation of the dry extract, as described in the “Materials and methods” section.

### 2.1. Dry Extract Characterization

The flow and compressibility properties of the obtained dry extract were determined according to the specifications of the European Pharmacopeia 9th ed. monograph, by measuring the untapped and tapped density and calculating the Carr’s index (CI) and Hausner ratio (HR).

The registered mean values of the untapped and tapped density were 0.48 g/mL and 0.56 g/mL respectively. The HR of 1.16 and CI of 16% described good flow characteristics of the final dry extract.

The final moisture content of the obtained dry extract was 2.72%. The TPC of the 8 samples withdrawn during the extract’s spraying phase are presented in Table 1. Based on the obtained results, the dry extract proved to be three times more concentrated in polyphenols compared to the liquid one.

The fluid extract’s concentration of 1.88% represented 1.88 mg gallic acid/100 g dry herbal material, but taking into consideration the fact that the dry herbal material/solvent ratio was 10%, the liquid extract’s concentration of 1.88% could also be expressed as 1.88 mg gallic acid/1 mL fluid extract. The concentration of the final dry extract obtained was 469.26 mg of gallic acid/100 g of dry extract (according to Table 1). Given that the fluid extract registered a density of 0.9595 g/mL, the 360 g of sprayed fluid extract represented 375 mL, the obtained 469.26 mg of gallic acid/100 g dry extract corresponding to 5.756 mg/mL fluid extract. Based on the fact that the 375 mL of fluid extract contained 6.768 mg gallic acid/mL fluid extract (according to the 1.88% gallic acid extract’s concentration mentioned above), the overall process yield could be calculated. A high yield of 85% was registered, value reflecting an efficient process, which therefore allowed us to obtain a concentrated dry extract, with a minimum loss in active principles.

### 2.2. NIR Spectra Analysis and Method Development

A NIR method has been developed for the *in-line* assessment of the TPC of the dry extract.

Firstly, all the acquired spectral data was imported into the SIMCA Multivariate Data Analysis (MVDA) software, in order to develop the spectral prediction model. A 2nd Savitzky–Golay (SG) derivative, with a quadratic polynomial order, was further applied to remove baseline effects and linear trends. This pre-processing technique also allowed the smoothing of the spectra and the improvement of the overall resolution of the information extracted from the spectra [35].

**Figure 1:**
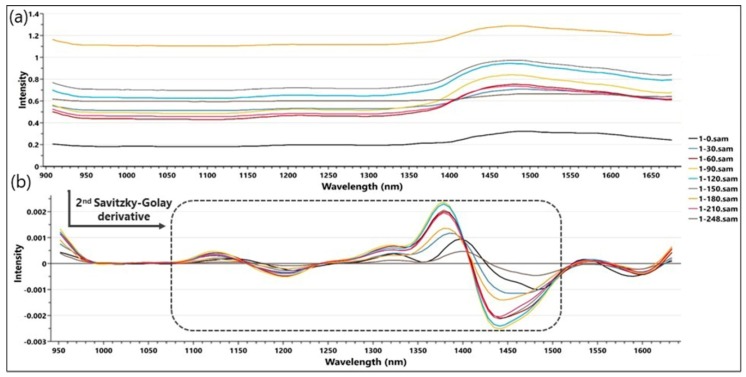
The raw (**a**) and 2nd derivative pre-processed (**b**) spectra registered during the fluid bed process and used for the development of the spectral prediction model.

The raw reflectance spectra, as well as the spectra pre-processed with the 2nd SG derivative algorithm are illustrated in Figure 1.

**Figure 2:**
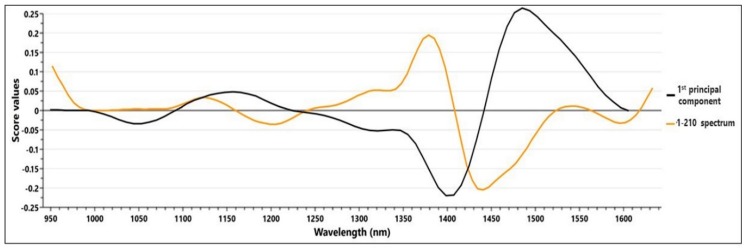
Loadings plot generated for the principal component analysis (PCA) model, overlapped with a preprocessed spectrum.

The highest spectral intensity variations can be observed between 1070 nm and 1520 nm, a domain which was further selected for the development of the Principal Component Analysis (PCA) model, which registered a R2X of 0.981 for just one principal component (PC). In order to summarize the relationship among the variables, the loadings plot of the PC was plotted in Figure 2, overlapped with a 2nd derivative preprocessed spectrum. The loadings score values registered two significant peaks, inversely proportional with the two intensity peaks of the spectra. This observation suggests that the high intensity regions account for the fluctuation of the most substantial variation of the samples properties, represented by the TPC.

The same spectral region chosen for the PCA’s model development has been further used for the development of the Orthogonal Partial Least Squares (OPLS) model. In order to do this, the 9 spectra (plotted in Figure 1), corresponding to the plain powder bed (t = 0) and the in-process withdrawn dry extract samples were correlated with the values representing their TPC assessed off-line by applying the UV-Vis reference method.

The calculated OPLS model included three factors: one predictive and two orthogonal ones. For the predictive fraction, high statistical parameters were registered, with R2X of 0.982 and Q2 of 0.951, showing good quality of the model. For the orthogonal (uncorrelated) fraction, just a low cumulated R2X of 0.077 was recorded, but which played an important role in the improvement of the overall quality of the model. The Root Mean Square Error of cross-validation (RMSEcv) corresponding to the three OPLS factors prediction model was 31.5 mg gallic acid/100 g dry extract, value which describes a good model, capable of delivering quality predictions.

**Figure 3:**
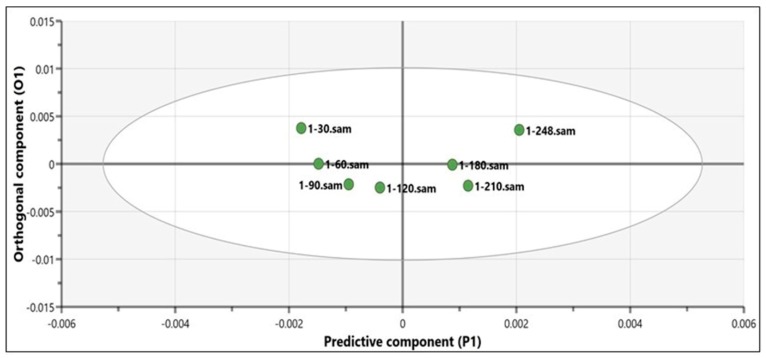
Scores scatter plot obtained from the orthogonal partial least squares (OPLS) analysis of the spectra corresponding to the 8 dry extract samples.

Further, the scores scatter plot of the OPLS model was calculated and represented. This plot summarizes the relationship among the model’s observations and allows the evaluation of their score value evolution. Figure 3 illustrates the predictive versus the first orthogonal component scores. It can be clearly noticed that the score values of the predictive component increase constantly starting with the first analyzed sample corresponding to the 30th spectra, to the last one corresponding to the 248th recorded spectra. In other words, the predictive component score values increase as the herbal extract adsorbing process goes on; i.e., the TPC increases.

The orthogonal component scores evolution did not vary much along the monitored process, thus suggesting that this component did not significantly reflect the properties of the product.

In order to perform an internal cross-validation of the previously developed method, the OPLS model was further applied during the ongoing process, in order to predict the TPC throughout it.

The values obtained by applying the reference UV-Vis off-line method, as well as the NIR predicted results for the same samples are presented in Table 1. The NIR predictions were relatively accurate compared to the measured ones, registering an average recovery of 98.7%. The correlation coefficient (R2) between the measured and predicted values was 0.994, describing a reliable prediction model.

**Figure 4:**
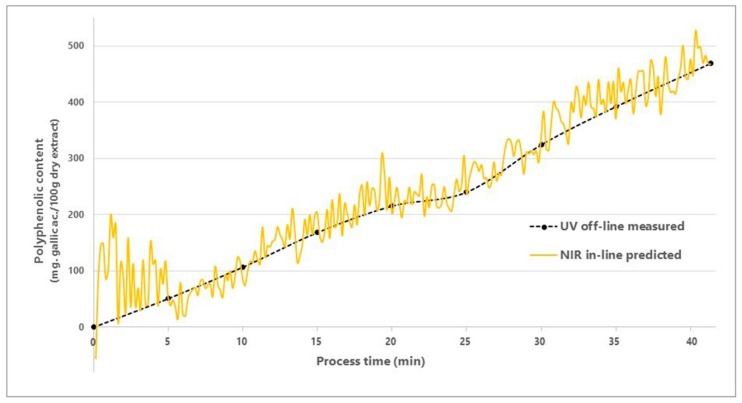
UV-Vis off-line measured vs. NIR in-line predicted total polyphenolic content.

For a better visualization, the TPC predicted values and the 9 calibration sample values were plotted over time, as illustrated in Figure 4. At the beginning of the spraying process, for the first 5 min, the TPC in-line predictions were not very stable, causing high prediction fluctuations, a fact which can be explained by an inhomogeneous product at this specific point. Moreover, for the first 5 min, the predicted TPC values were on average way higher than the reference, errors caused by a higher quantity of residual solvent present at the beginning of the extract’s spraying phase. As the process went on, the spraying and evaporation processes reached equilibrium, and their influence over the spectra became systematic. This systematic influence was assessed and eliminated with the use of MVDA during the development of the prediction model, resulting in the stabilization of the TPC predictions as the process went on and passed the first minutes.

Another observation made based on Figure 4 is that between minutes 20 and 25 the TPC increased at a slower pace compared with the rest of the process. This can be explained by the fact that, at some point, a small quantity of powder adhered to the NIR detector’s surface, thus impeding the recording of the spectra of the powder bed. In order to remove the obstructive powder from the surface of the NIR detector, the spraying rate has been reduced from 9 g/min to approx. 5 g/min. Consequently, after a very short period of time (about 30–60 s), the adhered powder was set in motion and removed from the detector by the fluidized powder bed.

The previously described approach, used in order to predict the TPC of the obtained dry extract in real time, enhances the control of the overall process, also enabling the identification of any modifications of the liquid extract’s spraying rate, which in this case represents a critical process parameter. Moreover, it allows the establishment of the process’s end point exactly at the moment when the product reaches the desired TPC concentration.

## 3. Materials and Methods 

### 3.1. Materials

The aerial parts of *A*. *genevensis* were harvested from wild populations from Cluj County, Romania, on July 2017, at full flowering stage. The voucher specimen of the studied plant was stored in the Herbarium of the Pharmacognosy Department of the Faculty of Pharmacy, Iuliu Hatieganu University of Medicine and Pharmacy Cluj-Napoca, Romania (accession number: AG-74). 

The air-dried natural product was grounded to a fine powder and extracted with ethanol, by using 50 g of plant material and 500 mL 70% ethanol, at room temperature. Lactose monohydrate (Tablettose 80) was purchased from Meggle, (Wasserburg am Inn, Germany), while the microcrystalline cellulose (Avicel PH102) was obtained from FMC BioPolymer, (Philadelphia, PA, USA).

### 3.2. Dry Extract Preparation and Characterization

The dry *A. genevensis* extract was prepared in fluidized bed, using a laboratory scale Aeromatic Strea 1 (GEA, Kirchberg, Switzerland) fluid bed processor, by adsorbing 360 g of ethanolic extract on a solid support composed of 30 g lactose and 90 g microcrystalline cellulose (120 g lactose-cellulose solid support). The powder bed was fluidized with an air flow of 7.5–12 m^3^/h, heated at an inlet temperature of just 30 °C, thus impeding the degradation of the product’s active principles.

Firstly, the powder mixture was preheated and homogenized for 10 min. Afterwards, the alcoholic extract was sprayed from the top of the expansion vessel, with a spraying rate of 9 g/min, through a 0.8 mm nozzle, the formed product being further dried in the same apparatus, over a period of 8 min.

A total of 8 samples of approximately 3 g were withdrawn during the extract spraying phase, through a sampling port, which allowed the sample collection without interrupting the ongoing process.

The total content in polyphenols was determined for the fluid herbal extract sprayed during the process, as well as for the 8 in-process withdrawn samples. The TPC was registered spectrophotometrically, using the Folin–Ciocalteu method, with some modifications, as previously described by Gavan et al. [13]. The absorbance was measured at 760 nm, using a JASCO UV-VIS spectrophotometer (JASCO, Tokyo, Japan). The calibration curve was obtained by using different concentrations of gallic acid solutions, the TPC being expressed as mg gallic acid/100 g of *A. genevensis* dry extract. 

The flow and compressibility properties of the obtained dry extract were determined by measuring the untapped and tapped density, by using a SVM tapped density tester (Erweka, Germany), followed by the calculation of the CI and HR [36]. 

The final moisture content of the dry extract was analyzed with the aid of an Ohaus MB45 (Ohaus, Parsippany, NJ, USA) humidity balance. Approximately 1 g of extract was placed onto the sample pan and subsequently dried at 80 °C, until the weight change was less than 0.2% over 10 min, the total percent of mass loss being automatically calculated.

In order to assess the performance of the developed process, the yield of the active principles adsorption was calculated using Equation (1).

(1) $$\;\mathrm{Yield}\;(\%)\;=\frac{\mathrm{Adsorbed}\;\mathrm{active}\;\mathrm{principles}\;\mathrm{content}}{\mathrm{Introduced}\;\mathrm{active}\;\mathrm{principles}\;\mathrm{content}}\;\times \;100$$

### 3.3. NIR Process Monitoring

The *in-line* monitoring of the fluid extract’s spraying process was performed using a MicroNir Pat-U spectrometer (Viavi Solutions, San Jose, CA, USA). This apparatus incorporates a Linear Variable Filter enabling its size reduction and facilitating the device’s direct attachment to the walls of the expansion vessel or to any other manufacturing apparatus, without using a fiber optic probe that could cause signal attenuation or dispersion. Therefore, the spectrometer is more stable and the measurements performed more reliable [37,38,39,40,41].

The apparatus was attached to the expansion vessel at the same height as the sampling port, so that the NIR detector could register the spectra by avoiding any interference with the process, as well as with the fluidized powder bed.

Spectra of the moving powder bed were registered in-line during the spraying of the liquid extract, without interrupting the ongoing process. The recordings have been performed continuously, at every 10 s, in reflectance mode, over the whole range of the spectrometer (950–1650 nm), with a resolution of 6 nm. Each spectra represented the average of 200 scans, recorded with an integration time of 7 ms per scan. All the above-mentioned parameters were set and controlled by the MicroNir spectrometer’s own JDSU Pro software.

### 3.4. Spectral Data Analysis

In order to extract the desired information from the large amount of in-line registered spectral data, the SIMCA 14.0 (Sartorius Stedim, Umea, Sweden) software was used to perform the necessary multivariate data analysis.

Firstly, a PCA was performed in order to get an overview of the gathered spectral data, as well as to identify the spectral domains which are specific to the changes related to the polyphenolic content. 

Secondly, an OPLS model was developed for the prediction of the TPC. This kind of model separates the X-specific spectral systematic variation into predictive and orthogonal (uncorrelated) fractions. An optimal number of OPLS factors was chosen based on the highest fraction of X variation modeled in the component (R2X), on the fraction of Y variation predicted according to cross-validation, by using the X model (Q2) and low RMSEcv and, in the same time, by avoiding the overfitting of the model [42].

The previously developed OPLS model allowed the *in-line* monitoring of the loading of the active principles on the solid support, thus enabling the control of the process and the establishment of the end point of the spraying process exactly at the moment when the product reached the desired concentration in active principles.

## 4. Conclusions

In the current study a manufacturing method for obtaining dry *A. genevensis* extracts by adsorbing the fluid extract onto an inert powder support, in a fluid bed process, was described. Moreover, an innovative NIR *in-line* polyphenolic content quantification technique was successfully developed. 

The obtained results showed that the liquid extract’s adsorption progressed smoothly throughout the process and that an increased quantity of active principles could be incorporated in the final product. Based on the obtained results, the dry extract proved to be three times more concentrated in polyphenolic compounds compared to the liquid one.

The method described in our study enables the monitoring of the technological process through the real-time quantification of the TPC, thus allowing the establishment of the process’s end point exactly at the moment when the desired TPC concentration has been reached. The novelty of this study resides in the fact that, to the best of our knowledge, no such method has been previously described.

## Figures and Tables

**Table 1:** Measured and near-infrared (NIR) predicted dry extract properties.

Sample	Spectra	Process Time (Min)	Reference UV-Vis Off-Line Method (mg Gallic Acid/100 g Dry Extract)	NIR-Chemometric In-Line Method (mg Gallic Acid/100 g Dry Extract)	Recovery (%)
1	30	05′	50.94	54.67	107.33
2	60	10′	106.79	86.93	81.40
3	90	15′	168.58	184.30	109.33
4	120	20′	215.75	203.30	94.23
5	150	25′	240.44	237.16	98.64
6	180	30′	324.81	340.44	104.81
7	210	35′	392.49	370.93	94.51
8	248	41′	469.26	468.05	99.74
